# The Hospital. Nursing Section

**Published:** 1904-05-07

**Authors:** 


### The Hospital.

### Nursing Section.

Contributions for this Section of "The Hospital" should be addressed to the Editor, "The Hospital" Nursing Section, 28 & 29 Southampton Street, Strand, London, W.C.

No. 919.—Vol. XXXVI. SATURDAY, MAY 7, 1904.

### Notes on News from the Nursing World.

### NURSING THE SICK AND WOUNDED IN JAPAN.

The details of the arrangements for nursing the sick and wounded in Japan, given in another column, which we owe to the courtesy of the President of the Red Cross Society of Japan, will be read with interest in all parts of the British Empire. It will be seen that they are of a very complete character, and attest the foresight of the managers. They also prove that foreign help is quite superfluous; and, best of all, it is evident that wounded Russian prisoners may depend upon receiving from Japanese nurses precisely the same skilled and devoted attention which is given to their own countrymen. A private correspondent in Tokyo under date of March 28th also mentions that a number of Japanese Red Cross Nurses and doctors have gone to Korea. In some places they have established temporary hospitals, and in others have made use of mission hospitals for the purpose of nursing the wounded. The nurses, our correspondent says, seem very capable in every way. The only evidence of war in the capital appears to be the passing through the streets of some of the troops. As in London, the newsboys are constantly running up and down with their "special editions."

### AN UNNECESSARY QUESTION IN PARLIAMENT.

Dr. Farquharson, who has charge of one of the State Registration Bills in the House of Commons and who should be well acquainted with what is passing in the nursing world, asked the Secretary of State for War on Monday evening "whether he had been able to effect any improvement in the pay and position of the Army nurse." If Dr. Farquharson had read the new regulations of Queen Alexandra's Imperial Military Nursing Service which, under the head of "Popularising Nursing in the Army," appeared in our issue of April 16th, he would have saved Mr. Arnold-Forster the trouble of answering his question. The Secretary for War stated that "a Royal Warrant would shortly be issued under which the increased salaries are fixed," and on Wednesday it was accordingly issued, but we set forth, three weeks ago, the full details of the improvements which have been made in the pay and position of the Army nurses.

### POOR-LAW NURSING AND RETROGRESSION.

At a meeting last month of the Executive Council of the Association of Poor-law Unions of England and Wales, the following propositions were adopted by large majorities:—"That the minimum age for commencing training should be 18 years; that 'qualified' nurses as distinct from fully-certificated nurses should be recognised, and that an infirmary without a resident medical officer may yet be a training school for nurses." Fortunately, these retrograde views cannot be put into practice without the knowledge and consent of the Local Government Board. But the proposition in favour of the "qualified" nurse reminds us that no action has yet been taken with reference to the report of the Departmental Committee. While strongly objecting to the creation of the "qualified" nurse, we think that the report should not continue to be treated as non-existent, and we are glad that Mr. Long was able to assure Mr. Schwann, M.P., last week, that he "hopes shortly to issue a general order."

### DEATH OF AN ENGLISH NURSE FROM PLAGUE.

A member of the nursing staff at Johannesburg Hospital has, we regret to learn, died from plague contracted in the performance of her duty. Miss Blake was one of the three nurses who, on the outbreak of the disease, immediately volunteered their services, and was employed at the Rietfontein Lazaretto, where the plague patients are nursed. At a meeting of the Johannesburg Town Council, it was decided to place on record the appreciation of that body of the self-sacrifice manifested by Miss Blake. The Mayor and other members of the Council expressed their sorrow that her life should have been lost, and the sad event has provoked a universal expression of sympathy in Johannesburg. The deceased nurse had many friends in England, who, while admiring her devoti n to duty, will be grieved that she has fallen a victim to the terrible malady which she volunteered to face.

### UNCERTIFICATED MIDWIFERY IN THE SISTER ISLAND.

Ireland is by no means behind the times in training women to be competent midwives, and the need for them was strikingly illustrated the other day at Belfast. An inquest was held concerning the death of an infant child. The mother gave evidence that she was attended in her confinement by an uncertificated nurse, who proceeded, after having washed the baby in whisky and water, to give it a spoonful of castor oil! The poor little mite died, and the doctor who was called stated that the dose was excessive and should not have been administered thus early. The Coroner expressed himself as astonished that such gross ignorance should still exist on such subjects, and asked the "nurse" whether she ever read the papers? Her reply was a decided negative, but she added with a touch of reproof in her voice to the questioner "I read the Bible!" It does not appear whether she intended to insinuate that she believed she found any grounds for the treatment pursued within the leaves of the Sacred Book, but she evidently considered that as long as she attended to her religious duties it was unnecessary to have any real knowledge of her secular obligations. We are not surprised that the jury returned a verdict condemning the impropriety of unskilled persons assisting at confinements.

### THE RELIGION OF NURSES.

An extraordinary motion is to be submitted to the Chorlton Board of Guardians, Mr. W. Ramsden intending to ask the Board to resolve that, "in all future applications for appointments, candidates be requested not to state their religion." The explanation given for this proposal is, that of the applicants for the post of superintendent nurse at the hospital 95 per cent. stated, unasked, that they belonged to the Church of England; and Mr. Ramsden alleges that several candidates for the vacancy were passed over simply because they did not name religion. However this may be, we think that any rule which suggests that religion is not a matter of much consequence is a serious mistake. Applicants for vacancies should not be chosen or rejected because they belong, or do not belong, to a particular church or denomination; but it is possible to push the undenominational principle too far in regard to nurses. If it became the custom for them not to name religion in their applications for appointments, the impression might soon obtain that it was not considered necessary that they should have any.

### NURSES' EXAMINATIONS AND CERTIFICATES.

The pass list of the nurses at the Central London Sick Asylum at Hendon shows that all the candidates passed their final examination this year, and that the minor probationers were equally successful. This is very satisfactory, and so is the report of Dr. H. P. Dean who examined the probationers at Poplar and Stepney Sick Asylum. The report states that the practical work was, as usual, very well done, and quite maintained the high standard of efficiency manifested by the nurses during the past few years. In awarding the certificates, Dr. Dean expressed the opinion that the matron's report as to the work of the nurses during their training, together with the results of the class examinations held by the teachers, should be considered apart from the examination for the certificate. He believes that if the nurses fully realised that the ward work and class examinations formed a definite part of the test of the final certificate, the conviction would tend to make them more attentive to daily routine.

### THE ANGLO-AMERICAN HOME AT ROME.

We hear that a maternity case in Rome has been offered to and accepted by one English nurse in Florence. This does not suggest that the Anglo-American Nursing Home enjoys the confidence which it ought to possess in the former city. But until it is managed by a strong and united committee it will continue to lack the general support which it might otherwise command. In consequence of the resignation of the matron being rejected by the managing committee at their meeting in January three of the members resigned, and it was anticipated that a special meeting of the general committee would be called to consider the question. There was, however, no legal obligation in the matter, and nothing has been done.

### NEWPORT GUARDIANS AND THEIR NURSES.

A fortnight ago we published an account of a remarkable state of affairs in Newport, Monmouthshire, showing that two candidates who were in succession appointed charge nurse at the workhouse hospital declined the appointment without giving their reasons. At the usual meeting of the guardians on Saturday the nursing question was brought up by an inquiry as to the strength of the staff and the number of recent changes. The clerk to the guardians said that the staff consisted of a superintendent nurse, three charge nurses, and eight probationers; and that since 1901 six charge nurses had been appointed, of whom three were still in the service of the board. There is nothing unusual in this record, but it appears that two months ago a probationer declined to continue her course of training at the institution, and would not state her reasons. This, coupled with the refusal, on April 16th, of the other two nurses to enter the service of the guardians, suggests, as the Rev. A. J. Jenkins urged at the meeting, that there is something which requires to be explained. Mr. Jenkins proceeded to suggest that the Chairman of the Visiting Committee should take a little more power into his hands, "and have a straight talk with those who ought to have the welfare of the institution at heart instead of their own little dignity upon which they tried to stand." Mr. L. S. Abrahams affirmed that the candidates who had refused to serve had been told "glaring untruths." Mr. Charles West thought that there had been a mistake in drafting the advertisement which appeared in The Hospital, and that the appointment should have been described as that of a staff nurse. Other speakers denied that the minds of the candidates had been "poisoned" by one of the lady guardians, but the lady guardians themselves maintained silence.

### A WISE DECISION AT TRURO.

Under the stress of great temptation, the committee of the Truro District Nursing Association have come to a very wise decision. Mr. Alderman Dorrington, who, when he was ill, found it difficult to secure the services of a trained nurse, offered a freehold house in the city to the Association, on the condition that they would engage a nurse to be paid by those who employed her. He also hinted at the probability of endowing it later on. But, while cordially acknowledging the generosity of the offer, the committee of the Association have reluctantly refused it "on account of the financial and other difficulties involved in taking up what would practically be a new branch of work, distinct from that coming within their province." If it can be found that there is sufficient work for a private nurse in Truro, we dare say that one might be easily induced to settle in the pleasant Cornish capital. In any case, however, it is satisfactory that the District Nursing Association have declined to attempt a combination which is always better let alone.

### OPENING OF A NURSES' HOME AT CHATHAM.

On Thursday last week the Countess of Darnley opened the Nurses' Home in Manor Road, Chatham, which has been erected by public subscription as a memorial of Queen Victoria. It is a two-story house of red brick with stone dressings and slated roof. On the ground floor are the superintendent's sitting-room, the "district-room," a general sitting-room, dining-room, kitchen, and usual offices. There are seven bedrooms on the first floor, also a box-room, a well-arranged bath-room, with modern equipments. The total cost of the building was £1,200. In declaring the building open, Lady Darnley dwelt upon the great interest which Queen Victoria always took in the nursing of the sick poor, an interest which was also manifested as keenly by Queen Alexandra. Among the guests was Miss Peter, who received a cordial welcome.

### DISTRICT NURSES AND COOKING.

On the afternoon of April 26th, the nurses of the neighbourhood of Taunton met at the Grange, Kingston, for a demonstration of invalid cookery by Miss May Joseph, who holds a first-class certificate from the Gloucester school of cookery. The demonstration was given in a cottage room, in a range such as would be met in the ordinary work of a district nurse. Everyone listened with great interest to the extremely well-arranged and practical lesson. After tea the nurses gathered flowers in the grounds of the Grange and returned to Taunton in various carriages as the shades of evening fell.

### NURSING IN BOMBAY.

A short time ago an attempt was made to establish "a nursing service for the Presidency of Bombay," and a small committee composed of the various nursing associations and Government officials was formed. But as there has been no meeting the presumption is that the project has been abandoned. There are obvious difficulties in the way of the proposed organisation. In any case, a combination between Government officials and independent associations is neither practicable nor desirable.

### A PAINFUL EXPERIENCE AT HENDON.

The nursing staff of the Central London Sick Asylum, at Hendon, have just had a painful experience, suffering, for the first time, the loss by death in the institution, of one of the minor probationers. Miss Annie Georgina Digby, who was only 22 years of age, entered the sick asylum in November last, and was taken ill, on April 7th, with suppurative pericarditis. Her condition was serious from the outset, but everything possible was done for her by the medical and nursing staff until the end. On the evening of the 23rd she was operated upon, and obtained a little relief. But a few hours later she passed peacefully away. Prior to the funeral at Wood Grange Park Cemetery, on Wednesday, last week, the body was carried by four porters into the entrance hall, preceded by the chaplain, and followed by the whole staff with the relatives. A short service was held in the hall before the coffin, covered with beautiful flowers, was conveyed to the cemetery. The managers sent an anchor of violets and primroses, the nursing staff from Cleveland Street a handsome cross, and the staff at Hendon contributed a cross of perfect white flowers to cover the coffin as a token of sympathy and sorrow.

### PAYING THE PIPER AT GRANARD.

At the audit of the half-yearly accounts of the Granard Union last month the Ratepayers' Association objected to several sums paid for the maintenance of special nurses in a hotel in the town, and for the salaries of special nurses, on the ground that such payments were unnecessary. The representative of the Association urged that there was ample accommodation in the workhouse for the nurses, and that the guardians should have appointed a permanent trained nurse when the medical officer suggested such a course. The total sum, which it was contended had been wasted was £95 4s. 2d. An attempt to answer this charge was made by the Chairman of the Board of Guardians, but the representative of the Association said that the Chairman had merely proved the case against the Guardians. The Local Government Board auditor, however, declined to hold that the money had been misapplied, whereupon he was asked to put his reasons in writing, and promised to do so. As the auditor admits that the Association can appeal to the Local Government Board, or to the King's Bench, we hope that they will do so. The issue is one of great and general importance.

### ONE NURSE TO 64 PATIENTS.

The efforts of some of the Guardians of the Eton Union Workhouse Infirmary to prevent the improvements in the nursing which have long been necessary have been defeated, and at the last meeting the report of the committee in favour of the appointment of two additional nurses, including one superintendent, was carried by a majority of eleven to four. But the spirit that kept Sairey Gamp flourishing still lives under the shadow of Windsor Castle, and one of the Guardians had the courage to declare on behalf of himself and some his colleagues that they thought pauper assistance better than too many professional nurses. This was after Dr. Newman had protested against "having one nurse to 64 patients," and Dr. Meggs had pointed out that the Local Government Board requires one to every 15 or 20 patients.

### PORTSMOUTH WORKHOUSE INFIRMARY.

On Monday the 25th, and on Saturday the 30th of April, the nurses of the Portsmouth Parish Infirmary underwent their annual written and oral examinations, the examiner being as usual Dr. Bryant, of Guy's Hospital. Of the 34 nurses who presented themselves 32 passed. Nurse Noad was the first of the third year's group, Nurse Oakes of the second, and Nurse Rosengrave of the first.

### DISTRICT NURSES WANTED IN COLLIERY VILLAGES.

From South Wales we learn that there is a pressing need in colliery villages for the employment of district nurses. It is said to be quite a common practice in the homes of the residents of these villages to hold prayer meetings in houses where a member of the family has died from an infectious disease; and many lives are believed to be sacrificed because there is no one able to make and apply a poultice, or provide jellies and articles of diet which would tempt a patient to eat and thus facilitate the stage of convalescence. Where the district nurse goes these things are altered. The duty of making provision for the needs of the poor people in the colliery villages seems primarily to devolve upon those who could not make money out of coal without the assistance of the workers.

### The Nursing Outlook.

" From magnanimity, all fear above;
From nobler recompense, above applause,
Which owes to man's short outlook all its charm."

### THE TWENTIETH-CENTURY MIDWIFE.

Nothing better indicates the coming status of the midwife than the fact that Dr. Watson has thought well to produce a substantial and most thorough text-book for her use. There are many books wrongly termed "complete"; but really Dr. Watson seems to have a right to use the term, for from title-page to index there is nothing missing, and, as he truly says in his preface, he has always preferred "too much" to "too little." And when we look at the regulations issued by the Central Midwives Board, and note the advertisements for inspectors of midwives, it becomes evident that in future we shall equal continental countries in our arrangements for this ancient and honourable employment for women.

Down to the eighteenth century accoucheurs were practically unknown, and the midwife always attended confinements, even in the Royal Family. Indeed, it used to be regarded as a crime for a man to take these cases, and it is on record that in 1522 a man named Werth was burnt at Hamburg because he dressed himself as a woman and attended a case.

Therefore, we are only regaining a lost stronghold when we ask that women should undertake this essentially womanly work; and we are merely protecting the mothers of the nation when we require that our midwives should be thoroughly trained and fairly intelligent, and that they should rather know too much than too little, that they may the better understand their responsibilities.

The hospitals are fast coming into line with the new state of affairs, and conforming to the regulations of the Central Midwives Board. At the General Lying-in Hospital in Lambeth Dr. Robert Boxall has undertaken extra lectures for the future, and the authorities are busy adapting a house opposite the hospital as a home for district-trained nurses and midwives. The plan of keeping those working in the wards entirely separate from those working in the district seems excellent, from the antiseptic point of view. There are no cases so terrible in the way of dirt and vermin as those to which a "charity" midwife is sometimes called in a London slum. Then the fees are to be rather lower for those who brave the dangers of the district—18 guineas as against 25 guineas for the midwives, and 9 guineas as against 12 guineas for the monthly nurses. Of course it is desirable that the midwife who is going to take up a town district should train in the district, and learn the great art of "going without" in the way of appliances. There is a tale on record of a case in Southwark where the only utensil for all purposes, including washing the child, was a beer can. Moreover, only experience can teach how to pack a bag so that it shall contain what s absolutely necessary, and yet not be too grievous a burden. It may be all very well for the midwife who looks forward to a "high-class" practice to train mainly in the wards, where everything is to hand, and cleanliness reigns supreme, and the physician is within call; but the "all-round" woman who intends to seek a country practice will probably do well to get as much outdoor work as she can.

The period of training is still rather short bearing in mind that a two years' course is common on the Continent: in most hospitals it is still only three months for a midwife and two months for a nurse. Quite a number of scholarships and "helps" towards training have been founded of late, and with their aid it should have been possible to give longer experience. The fees may sound rather heavy at first, but when it is considered that they include board and lodging and instruction, and generally washing as well, two guineas a week is not excessive. A library of good books, models, and "bones" and class instruction, as well as lectures, are always provided, and if the improved status of the midwife is making some of our readers turn towards the work, we would suggest that it is better to invest a good round sum in a good all-round training, and that in midwifery, as in all other walks of life, a good start is half the battle.

A deputation of medical men has recently approached the Principal of University College, Cardiff, and proposed that the College should add to its medical school a department for the theoretical training of midwives. The Principal received the suggestion favourably, and said that if the County Councils of Cardiff, Glamorgan, and Monmouthshire would help, they would gladly form the training centre for South Wales. All this shows the forward tendency.

The full force of the Midwives Act will not be felt for some years; it has been graciously tempered to the old-fashioned and often highly-skilled worker; but the young woman entering on an important calling should not shelter herself behind any temporary expedients, or try to creep through her examination with the least possible amount of cram. We agree with Dr. Watson that no training can be too complete for the midwife of the future, and that every effort should be made to urge her on to a high ideal of the knowledge necessary in her employment. The examination of the Obstetrical Society, and of all other societies and schools of midwifery, is bound to grow more severe, and to be fitted to pass it with ease, and then to practise it with certainty and skill, should be the aim of everyone now taking up midwifery.

### Lectures Upon the Nursing of Mental Diseases.

By Robert Jones, M.D.Lond., B.S., F.R.C.S.Eng., M.R.C.P.Lond., Resident Physician and Superintendent of the London County Asylum, Claybury.

LECTURE XI.

The (2) *special* duties of the mental nurse depend upon the class of cases she is called upon to supervise, and the classification of insanity given in a former lecture will be adhered to, viz.:—

(*a*) Those who are depressed—cases of melancholia.

(*b*) Those who are noisy and excited—cases of mania.

(*c*) Those who have fixed ideas—called cases of delusional insanity.

(*d*) Cases of weak-mindedness following long-continued depression or excitement—cases of dementia.

(*e*) Cases of general paralysis with insanity.

(*f*) Cases of epilepsy with insanity.

(*g*) Cases who from birth have been deprived of their intelligence, such as those suffering from idiocy and imbecility.

In cases of (*a*) *mental depression*, the nurse should endeavour to cheer the patients by kindly sympathetic conversation and conduct. She should divert their thoughts from distressing fancies by suggesting pleasant topics and by avoiding the subject of their delusions. This can often be done by inducing them to engage in some active and light occupation, or some form of amusement which they can share with others. It is found in asylums that, in the treatment of the insane, the full complement of mental reaction is best obtained between the two extremes of excitement and depression, and this is secured by bringing the two extremes together. Thus cases of marked depression do best in the presence of those of a more excitable temperament, and in a well-arranged classification of patients in an asylum cases of mania and cases of melancholia are for this reason associated in the same ward. The one provides the complement to the other. It is well for the nurse to remember that suicidal attempts take place most often among cases of melancholia, and that among these "the means to do ill deeds makes ill deeds done." The appearance of sharp cutting instruments may prompt an unbalanced, depressed patient to a rash act. For the same reason open windows must be secured, bath taps must be locked, strings on the dress, or boot laces and garters must be removed, and the food must be cut up so that it can be taken with a spoon. Many cases of melancholia are exceedingly restless, and will appear to the nurse more like the exaltation of mania. Such patients with undue motor excitement should never be away from the constant care of the trained nurse. The patient should be carefully watched in regard to taking food, but the nurse can only coax and stimulate, and if her encouragement fails, the doctor will probably administer nourishment forcibly, either by the nasal or stomach tube or—when the food is pre-digested—by the rectum. Cases of melancholia are often worse in the early morning than in the evening, being thus unlike cases of nervous depression, which form is called neurasthenia, and nourishment in cases of depression is especially required. It is always desirable to give cases of melancholia some liquid nourishment in the night hours to avoid and counterbalance this early morning exhaustion and depression. Massage and Turkish baths may be ordered to stimulate the skin, and when exhaustion has been overcome the patient should be encouraged to take gentle exercise in the open air. To prevent cases of melancholia passing into dementia, general electrical stimulation is often prescribed, which the nurse should know how to carry out. Care should always be taken in bedding cases of depression; their bed and bedding, as well as their own persons should always be examined to prevent their secreting anything that may prove hurtful to them, and on no account is the word of a patient to be taken for granted that there is no means to do bodily harm secreted about the person without verification—not even are those who are convalescing to be trusted without confirmation.

In cases of (*b*) *mania*, when patients are noisy, excited and turbulent, they should, so far as is possible, be soothed by persuasion and judicious management, kept from annoying other patients, and provided with a healthy outlet by muscular exercise—a method of treatment which is invaluable in severe and long-continued excitement. In some cases much exhaustion results from this condition, and the recumbent posture is retained. Such patients are kept in bed for days together and forcibly fed if they refuse food or take it in insufficient quantity. Generous feeding is especially necessary for these cases as the exhaustion is very marked. At times such patients are sent for vigorous out-door exercise, for they put forth fewer efforts at violence and restlessness in the freedom of a garden or during a walk than when kept in bed. In such cases warm baths may be used continuously for many hours, and if the nurse is able to control this it is often a valuable means of procuring sleep. The "wet pack" is at times used to control excitement. This is done by immersing a sheet in warm water, then, after a moderate wringing, placing the sheet lengthwise on the bed, from the head to the foot of the bed, having previously stripped from it all clothing except the lower sheet. The patient is then rolled, with the arms to the sides, in the wet warm sheet, taking care that this is not too tight, the head only appearing out of the sheet. The bedclothes are replaced over the patient, who is carefully and continuously watched by the medical officer. The patient is kept in this state from one to three hours, according to the mental and bodily conditions. The same procedure with a dry sheet is termed the "dry pack." When the patient is taken out of the pack, the skin is sponged over with warm water, and the patient placed ordinarily in bed. Especial care must be taken in the use of this treatment, which is considered to be a form of restraint, and all particulars of its application must be reported to the Commissioners in Lunacy, if the patient is certified, and its necessity for an uncertified case—except for purely medical reasons, such as dropsy, or suppression of urine—would be a strong argument for placing the patient at once under certificates. In chronic cases of mania, the general indications for the nurse are to feed, clothe, and safeguard the patient, and to divert the mind with regular occupation or amusement. The indications for treating cases of (*c*) fixed ideas (*delusional insanity* as this form is called) are, not to argue with them, but tactfully to divert and give them occupation. More, however, will be said on this point when dealing with the personal duties of the mental nurse.

In cases of (*d*) *dementia*, the nurse has, more or less to think for the patient in everything. The object is custodial. She has to see that they are properly dressed and undressed, that they are sufficiently clothed and protected from exposure to cold and other danger, and that they get a due amount of outdoor walking exercise Many of these cases are very trying to nurse, as their habits are objectionable, and they are liable to hurt themselves without complaining.

Constant attention to cleanliness must always be observed, and all demented patients are better up and about than in bed, so long as this is possible, for confinement to bed may mean bed sores and premature death from low forms of septic inflammation. The nurse should watch for any changes in the patient and report the same at once to the doctor, as frequent physical examination is necessary to detect the insidious onset of lung, kidney, or heart affections; or even to detect such accidents as fractured ribs so apt to occur in this self-neglected class, and which may otherwise escape notice and be overlooked. Regular hours of meals, warmth, and proper sleep are also essential for this class.

In cases of (*e*) *general paralysis with insanity* the treatment, so far as the nurse is concerned, is to prevent the patient doing harm to others, to keep the patient clean, and that there should be adequate body warmth by night and by day. Lastly, but chiefly, that every precaution should be observed as regards the taking of food. General paralysis is incurable, and its duration is a matter of time; long—often many years—in the case of female patients. Speaking generally this form of insanity is a disease of men, who fall victims to it at least three times as often as women. These patients, towards the end of their disease, are to the nurse objects of special solicitude, for they eat voraciously and rapidly, and will often steal the food from the plates of other patients, cramming it into their mouths, and there is a great risk of choking as the advancing paralysis causes loss of the power of swallowing, also of liquid nourishment entering the lungs and causing fatal broncho-pneumonia. In such cases all food is given slowly and in a soft form, such as minced meat, minced vegetables, milk, custard, and the soft portions of bread well soaked in milk or tea. These patients often eat leaves, grass, pieces torn off their clothes, bits of rags they pick up, or the corners of the sheets when in bed. The nurse must be very careful to prevent this or the patient runs a considerable risk.

(*To be continued.*)

### The Nursing of the Japanese Wounded.

BY THE PRESIDENT OF THE RED CROSS SOCIETY OF JAPAN.

The President of the Red Cross Society of Japan, Count M. Matsugata, writing from Tokyo under date of March 23rd, kindly gives us the following official details as to the arrangements, present and future, of the society for the nursing both at home and abroad of soldiers wounded in the war between Japan and Russia.

The organisation is divided into five departments, viz.:—I. Department for general emergencies at the seat of war; II. For the transportation of patients; III. For the nursing of patients on boats; IV. For the nursing of patients in hospitals; and V. For the supply of necessary materials. Each department is quite separate from the other, and they all act entirely in accordance with instructions given them by the Ministers of the Navy and of the Army respectively. The nursing arrangements already in working order are as follows:—

I. Detachments each prepared to meet and take charge of 100 patients consist of two physicians, one apothecary, one clerk, two directors of the female nurses, and 20 female nurses.

II. Detachments each prepared to meet and take charge of 30 patients consist of one physician, one clerk, two directors of male nurses, three directors of the transport division, 120 officers of the transport division, and three male nurses.

III. Detachments each prepared to meet and take charge of 100-200 patients consist of one manager, four physicians (including their superintendent), one apothecary, two clerks, two assistant apothecaries, two directors of female nurses, two directors of male nurses, 20 female nurses, and 20 male nurses. In addition to these there are smaller boats with half the administrative staff.

IV. This department consists of hospitals which are temporarily constructed at those stations where patients are carried in.

V. Detachments are located at a convenient place for distribution at the seat of war, and consist of one manager, one apothecary, and one clerk to distribute necessaries.

Up to the end of March there were 98 detachments ready for general emergency, chiefly in the hands of female nurses, 18 detachments for the same in the hands of male nurses, three detachments for the transportation of patients, four detachments attached to nursing boats, and one detachment for the supply of necessaries. As many more detachments as are required can, however, be at once despatched as soon as it is found that they are needed. Amongst the hospital ships, two, the *Hakuai* and the *Kosai*, were engaged in the last Chino-Japanese war, and in consequence of the experience then gained they have been much improved, rendered more suitable for the purpose and more comfortable, and are largely in the hands of those who then proved their suitability for the work. No engagements of much importance having taken place very early in the war, the nurses have had comparatively few patients of their own nationality to tend, but have had charge of several Russian sailors wounded in the naval battle of Chemulpho, off Korea. Having obtained the permission of the British Minister in Korea, these men were taken into a hospital established in a house attached to one of the English churches in Korea, and were there treated under the direction of the special committees of the Japanese Red Cross Society. How kindly and successfully they were nursed back to health is testified by the letters received from those who experienced treatment at the hands of the Society's organisation. Owing to the severity of their wounds many had resigned themselves to a speedy death, but as a result of the care bestowed upon them the majority have pulled round. After a little time it was found advisable to transport the patients to more convenient and more comfortable quarters than it was possible to obtain at Chemulpho, so the temporary hospital there was closed and they were conveyed, twenty-two in number, by the ss. *Hakuai* to a hospital attached to a provincial district, Matsuyama, Iyo, Japan, where everything is up-to-date and the best procurable for the comfort and restoration to health of all who enter the hospital.

It was touching to note the attitude of the Russian patients as they were embarking at Chemulpho. Their farewell to the surgeons and the nurses who had attended them was more suggestive of the affection usually shown by beloved children to their parents than of the sentiments supposed to be entertained by one foe towards another. They are doing remarkably well at Matsuyama, and the hope of all concerned is that they may soon be quite restored to health and able to return to Russia and once more greet their parents, their wives, and their little ones.

### Midwifery: as Practised by Native Midwives in Fiji.

By the Hon. B. Glanvill Corney, Chief Medical Officer of the Colony.

*Continued from page* 69.

When a wise-woman, or so-called midwife, is in attendance on a case, she makes occasional digital examinations for the purpose of ascertaining the nature of the presentation, which is rarely other than normal. It is not usual to tamper with the membranes, and practically nothing is done until the birth of the child has been naturally effected. The first act of the midwife then is to clear its mouth of mucus by the not always gentle use of her finger, or by the nasty expedient of applying suction with her own mouth. It is not an uncommon thing for the midwife to force her index finger well back into the pharynx of the infant for the same object; and cases are on record in which fatal laceration of the parts with plugging of the glottis, has occurred during this manœuvre. It is nevertheless considered by native women a most essential item in the performance. There seems to be no definite understanding as to the precise stage at which the umbilical cord should be severed. They wait, however, until the child cries, or at least breathes; but the Fijians are ignorant of the fact that the blood circulates, and though some midwives have assured the writer that they knew there was pulsation in the cord it was quite obvious that they were not aware of its significance. When the child fails to cry the general practice is to seize the cord lightly between two fingers, or a finger and the thumb, on the placental side, and, by passing them along it towards the umbilicus, to squeeze the blood in it forwards in the direction of the child. They also have a trick or "dodge" of shaking a rattle near its face, in the hope of awakening it into life. This rattle is constructed of a bunch of dry, hard, mature cocoanut shells strung together; and in some parts of the islands its component parts are always ready to hand, being ordinarily used for conveying and storing fresh water, and may be seen lying in a corner of every dwelling. Nothing is known of the value or methods of artificial respiration in such cases, nor even of the efficacy of cold water or the familiar flip of a wet towel. In fact they have no towels, though the native bark cloth would serve the purpose equally well.

The custom in severing the *funis* is to measure it off from the umbilicus to the child's knee, and then cut it across with a sharp mussel-shell or splinter of bamboo or cane; it is cut *koso*—which means clean and square across—not jagged or scraped through, and not obliquely. Nowadays, scissors are generally used. It is never tied or knotted, nor is any kind of ligature, torsion, or pressure applied. Fijians never sever the cord by biting it, as is the case with some savage races of men, and with many of the lower animals. Native opinions are at variance as to whether hæmorrhage ever occurs in consequence of the omission to ligate the *funis*. The midwives deny that it does but there seems to be a general impression that it is a good thing to let out the *dra ca*—bad blood as they term it—remaining within it after the placenta has been shed from its attachment. Division of the umbilical cord without ligature is, in point of fact, not so unsafe a measure as may at first appear to those who have always been taught that to tie it is an indispensable precaution. The accumulated experience of obstetricians goes to show that in such cases the greater the length of the fœtal end left after division, the less is the risk of hæmorrhage; but a clean cut made transversely with a sharp instrument is, of course, more likely to allow bleeding to ensue than is an oblique or lacerated severance. Nurses will readily understand this if they have remembered their teaching on the structure of blood-vessels and the surgical pathology of hæmorrhage. After dividing the cord, the Fijian woman wraps the fœtal end in a small piece of *seavu*, which is native cloth, made by hammering out the inner bark of the paper mulberry tree after several soakings in water and sticking the strips together. This is then coiled down on the abdomen and left pretty much to chance; but the cloth is changed from time to time as it becomes soiled with the blood which oozes from the open ends of the veins of the cord and clots about its fibres.

As soon as the cord has been divided, an attendant re-moves the child, and washes it in water, not warmed, and without soap. At the same time a draught of cold water is given to the mother to swallow, with the view to stimulate the uterus to contract and get rid of the afterbirth. The prevalent opinion among native midwives regarding this process is that the placenta is generally extruded spon-taneously. Delay is, indeed, uncommon; and retention of the placenta is as rare as it is dangerous, and is the one serious contingency dreaded by native women.

In the hill country of Na Viti Levu, labour seems gene-rally to be more easy and expeditious than on the coast; yet, strange to say, the midwives of the uplands bear the reputation of being specially clever, and notwithstanding their less varied experience, they do appear to follow more orthodox methods than their sisters of the coast, amongst whom the Tongan influence has in some degree made itself felt. But, like the latter, they are entirely ignorant of the construction of the human body and of the functions of [illegible] several organs, its viscera, and parts. Their practice is con-sequently quite as empirical in the management or [illegible] management of child-birth as it is in matters relating [illegible] nursing in general illness and the care of children.

Inland, the midwives will introduce the hand—and [illegible] a hand as would appal an English nurse trained in asep[illegible] principles—to effect extraction of an adherent placenta, [illegible] one which, as often happens, having been cast off from [illegible] interior of the uterus remains lying in the fundus of a [illegible] and inelastic vaginal wall. On the coast they prefer [illegible] await its complete extrusion by natural means, [illegible] have a decided objection to giving manual assist-ance in this process; declaring that it is a danger[illegible] if bold practice, and one they neither understand [illegible] care to commit themselves to. In such cases, when [illegible] delay is considerable—the customary drink of cold wa[illegible] having been given ineffectually—herbal infusions are [illegible] administered. Local external applications of bruised [illegible] chewed leaves, in the nature of cataplasms, are also said [illegible] be used; but the excellent and safe expedient of stimulat[illegible] and compressing the uterus by means of the hand placed [illegible] the abdomen is quite unknown among Fijian midwives—[illegible] circumstance somewhat surprising in a nation of masseu[illegible] Fijian mothers do undoubtedly die sometimes from retain[illegible] placenta, and the fact that in such cases the midwife g[illegible] all the blame if she have perchance attempted any man[illegible] interference, is perhaps the reason why these ladies [illegible] generally unwilling to attempt it. The writer once extract[illegible] a placenta which had been retained in the uterus itself [illegible] seventeen days after the birth of the child, and the mot[illegible] did well. Its condition was then partly organised, se[illegible] fleshy, but quite hard and spherical; and anything more l[illegible] a schoolboy's cricket-ball in size, shape, weight, colour, [illegible] hardness would be difficult to imagine. Some Fijian wo[illegible] have gone so far as to state that many live in dread [illegible] pregnancy, simply through the popular fear of retai[illegible] placenta—ignoring all other contingencies.

(*To be concluded.*)

### One Day in a Children's Convalescent Home.

BY A NURSE.

7 A.M.—It is a lovely morning in early summer. Outside, there is the happy sound of birds singing, in the distance the noise of a hay-making machine, and the lowing of cattle; in fact, these and many other sounds indicate that Nature is well awake. Inside, in the children's ward, with its rows of cots, comes the sun, casting a subdued light, for the venetian blinds are still drawn, and it is only here and there that he can peep through with any show of brilliancy. All appears quiet, though several movements in the various cots seem to indicate that the occupiers are not contemplating further sleep.

7.15.—Footsteps are heard coming along the corridor, and someone enters the ward. It is Nurse. There is a lifting up of heads from the pillows, and a subdued stir in nearly every bed. Nurse draws up the blinds and opens the windows. In comes the sun, flooding the ward in a blaze of light; with him comes the fresh morning air bringing the scent of new-mown hay, and honeysuckle and wild roses. Nurse says, "Now, children, you may get up!" The sun and the breeze say the same, but in a more ancient language. In less than a minute, down go the cot-sides, and eight or nine people are scrambling out on to the floor, and into dressing-gowns, which they fling on with a fine disregard to the use of sleeves, and out trot these eight or nine boys and girls to have their morning wash, though perhaps the word "trot" is hardly suitable for all, as some go on crutches, and some hop along using the cots for support on their way. But everyone is not lucky enough to be able to go out to the bath-room to wash; there are several cots whose sides are still up. In one you will see Patrick; he has one of his legs in what he calls a "plint;" next but one to him is another individual of four who has had one leg amputated; he is just now hopping round his cot singing "Dolly Grey," only stopping to ask at intervals "when shall you wash me nurse?" Next to him is Mary, an empyema, rocking her pillow for a make-believe baby, and placidly waiting her turn to be washed. Opposite to her is Emma, in a double Thomas' splint, clasping a doll. These children are all staying in bed to have their various dressings done, but dressings do not come until after breakfast. When everyone is washed and the "getting up" people dressed (which is always a lengthy proceeding for all concerned), there is an exciting rush for chairs and a settling of the children who are up, at the breakfast table. After this important business is ended, behold this small and tidy company expectantly awaiting breakfast.

8.15.—Footsteps again along the corridor. This time it is not nurse but the maid, and to the supreme satisfaction of the awaiting company, breakfast arrives. Nurse says, "Sing your grace children." This ended, plates are handed round, then the bread and butter, then the eggs, and finally the mugs of cocoa, and everyone "sets to" and eats to the best of his or her ability. Breakfast finished and grace said the children troop downstairs to get on their garden shoes and hats, and off they go into the lovely sunshine and fresh air.

9.15.—It is now time for dressings, and instruments are being sterilised and lotions got ready, and the business of the morning begins. Patrick awaits the dressing of his knee like a Spartan, asking Nurse if she is going to "put the rag in with a pin!"—his idea of a plug of gauze and a probe. "Don't pin it much, only a lickle bit!" says the patient, watching the plug going in with intense gravity. Finally it is finished, and the gentleman dressed and carried off downstairs, to be strapped in a mail-cart and wheeled into the garden, his toilet being completed with a broad-brimmed straw hat, which he promptly throws on the grass.

10.30.—By this time all the dressings are finished and all the children are downstairs and out in the garden, the spinal carriages with their respective occupants are wheeled into the shade under the trees, and there is a shout of "Lunch!" from the boys, and a patter of running feet and the stump of crutches as everyone comes up to Nurse for biscuits and a drink of milk; then off they go again to play their various games.

12.30 P.M.—It is now time to get ready for dinner. The spinal carriages and mail-carts are wheeled in, and everyone is washed and "tidied" for dinner, and in a short time a clean and expectant party is sitting round the dinner-table waiting for sister. The dining-room is large and airy, with many windows through which one gets a glorious view of fields and wooded country. A cheerful noise is kept up the whole of dinner time, the entrance of the pudding being greeted with a subdued murmur of delight, because it is all jam on the top.

2.—Now comes to some of these small people the great event of the day, that is the drive in the pony-trap. Every one goes for a drive in their turn; this afternoon the happy four whose turn it is have been got ready, and are fitted into the trap and away goes a smiling party with one of the nurses to drive.

4.30.—The driving party has come back, the tea-table is brought out on to the lawn, the cake and bread and butter set out, and after singing grace the meal proceeds. Presently some one cries out "the organ man," and down the drive comes a man wheeling a barrel-organ, who plays all the well-known tunes, while the children finish their tea.

6.30.—Supper time, a small crowd of hot dirty little persons devour biscuits and drink milk, chairs are taken in, books and toys are collected, and sister and nurses starting in procession with their various helpless burdens, taken out of spinal carriages and mail-carts, proceed up the staircase to the ward.

6.45.—Upstairs in the ward. Such a noise! Comic songs being sung and shouted in varying keys by various small voices; here someone trying vainly to untie a knotted tape of a pinafore; someone else struggling out of a rather tight under-vest; but everyone more or less busy. Those who cannot be bathed are being washed in bed, and the remaining children are in the bath-room waiting their turn for the bath, after which ceremony they appear once more in the ward, wearing a very polished countenance and a dressing-gown doing duty more or less as a court train. All at once there is a rush of feet from the bath-room. Someone has refused to be bathed, and there is an exciting chase round the table, the pursued takes refuge under a cot, but is finally fished out amid the shouts of the onlookers, and carried off captive to his ablutions.

7.30.—Everyone is now tucked in bed, and peace and quietness reigns in the ward, there being no other sound but Sister's voice reading prayers. Then the blinds are drawn down, and after a cheerful chorus of "Good nights," everyone settles down quietly, as the first step towards a restful night.

### Where to Go.

GRAND MATINÉE CONCERT, Grosvenor House, Tuesday, May 10, at 3 P.M.—In aid of the rebuilding fund of Lower Brixham Church, Devon, in memory of the author of the hymn "Abide with Me."

### Metropolitan Nursing Association.

### ANNUAL MEETING.

PRINCESS CHRISTIAN presided at the twenty-eighth annual meeting of the Metropolitan Nursing Association, held, by permission of the Duke of Westminster, at Grosvenor House on Monday afternoon.

Among those present were Alice, Countess of Strafford (Patroness of the Association), Miss Hadden (superintendent), Miss Peter (superintendent of the Queen Victoria Jubilee Institute), Miss Amy Hughes (superintendent of the County Nursing Association), the Rev. and Mrs. Dacre Craven, the Rev. R. Peile, (Master of St. Katharine's), Mr. Henry Bonham Carter, Mr. Harold Boulton, Mr. F. D. Mocatta, and others.

The adoption of the annual report and accounts was moved by Mr. Henry Bonham Carter, who remarked that it was not to be expected, even it were desirable, that a great deal of variety should be shown in the reports issued from year to year by the Association. If the work went on steadily improving in its value and character, the committee were content; and this, he believed, was the case with regard to the record for 1903. Although, owing to new buildings in the neighbourhood, the character of the population had changed to some extent, yet the number of poor within the area covered was larger than could be adequately dealt with by the staff, and the Association continued to be a most valuable source of relief to the sick poor in the surrounding parishes. The statistics showed that 1,359 patients had been attended, of whom 136 were transferred to hospitals. This was important, because it indicated that the nurses had been instrumental in inducing that number of serious cases to undergo treatment by which alone they could be really benefited. Of the remainder, 897 were declared convalescent. Of the total number visited, by far the largest proportion (974), were women, 149 were men, and 139 children. A large number of patients (64) were suffering from cancer, a disease requiring very careful nursing, and here the value of the really trained hospital expert nurse was strikingly evident. The usual eminently practical courses of lectures had been given during the year, and 17 nurses had been trained for, and received appointments under, the Queen Victoria's Jubilee Nursing Institute. With regard to finance, the Association was able to close the year with a deficit of only £5, but new subscribers were urgently needed to take the place of those removed by death.

Mr. Harold Boulton, in seconding the resolution, spoke in warm terms of the value of the training given at the Bloomsbury Nurses' Home. The Jubilee Institute attached very great importance to the district training of its nurses. The fiction that a perfectly-trained hospital nurse was able at once to nurse the sick poor in their own homes, was not confined to the public; even nurses, until they had had a little practical experience, being sometimes rash enough to believe it. Mr. Boulton then threw out the suggestion that, owing to the shifting of the working-class population, it might be found desirable to move the centre of operations from its present premises; but whether this were so or not, he appealed to those present to make the excellent work of the Association more widely known, and to help it in its struggle for existence.

The report having been adopted, the Rev. R. J. Campbell moved a resolution pledging the meeting to support the Association in carrying on its beneficent work. This was seconded by Dr. Oswald Browne, who spoke from personal knowledge of the quality of the nursing, and commented on the number of visits, *i.e.* 28,467, paid during the year.

The resolution was carried. The Rev. Dacre Craven, in proposing the re-election of the Council, urged upon Boards of Guardians the wisdom of supporting the District Nursing Associations, and read a letter from one such body testifying to the high estimation in which the work of this Association was held.

Mr. F. D. Mocatta having seconded the resolution, it was carried, and a vote of thanks to Princess Christian for presiding, and to the Duke of Westminster for the use of the room, brought the meeting to a close.

### Everybody's Opinion.

[Correspondence on all subjects is invited, but we cannot in any way be responsible for the opinions expressed by our correspondents. No communication can be entertained if the name and address of the correspondent are not given as a guarantee of good faith, but not necessarily for publication. All correspondents should write on one side of the paper only.]

### A MATRON'S DIFFICULTY.

"MATRON" writes: I shall be glad if you will allow me a small space in THE HOSPITAL. I am a matron in a hospital of 54 beds, and should like to know if other matrons experience the same difficulty as I myself do when changing sisters and appointing one who has recently finished her training. She enters upon her duties with an idea that she is merely to give instructions, and not to do any work with the probationers. I myself as a sister felt it my duty to assist any nurse who wanted help with the patients and instruct new probationers myself as far as I was able; but each fresh sister I have thinks she ought not to do so much. My own nurses I have no difficulty with when appointing them sisters.

### FILLING A WATER-BED.

"PRIVATE NURSE" writes: In reply to "Nurse H.'s" query, there need not be much difficulty about her water-bed, even without the second bedstead, as the bed the patient is on is a wide one. I should draw her as far as possible to the right side of the bed. Then draw the bed (feathers) with the patient on it as far as possible to the left side, put chairs or some support, so that she may not be afraid of falling. As soon as she is on the inner edge of the feathers, and in about the middle of the bed, the water-bed should be laid on the free space, and filled with the help of a funnel, a jug, a few pails of water, and at least 10 quarts of boiling water. When sufficiently full and of moderate warmth, the feather-bed may be worked a bit farther off, and the water-bed moved towards the middle of the bed. A fairly thick blanket and a sheet should be put over it, and the patient drawn on to it on her own sheet, which can be rolled from under her when convenient.

### ROYAL NATIONAL PENSION FUND FOR NURSES.

MR. LOUIS DICK, Secretary of the Pension Fund, 28 Finsbury Pavement, writes: A paragraph recently appeared in one of the halfpenny daily papers from which the following is an extract:—

"Of the 353 hospital nurses who surrendered their interest in the Royal National Pension Fund last year 84 confessed it was to 'go and be married.'

"Mr. Louis Dick, when interviewed, expressed the opinion that the large percentage of marriages among nurses was not that they were more attractive than other women, but rather because men are mercenary.

"'A nurse who has put by some money in our Fund,' said Mr. Dick, 'brings a dowry with her. Nurses during their working life—that is, between 28 and 40—earn more than the ordinary working woman. After 40 they are not much use.'"

The following are the facts: On the day of the annual meeting a reporter called here and asked me for various particulars respecting the Fund, which I gave him; amongst other things I drew attention to the fact, which the chairman subsequently brought out in his speech, that last year while the number of nurses joining was larger the average age was higher and the amount of each individual policy smaller, and told him of the difficulty which the nurses about 40 years of age had in obtaining work, and that as years increased this difficulty became greater; this he has paraphrased into "after 40 they are not much use." In another place he says that the "large percentage of marriages among nurses is not due to the fact that they are more attractive, but because men are mercenary." Now this sentence taken by itself means nothing at all: to say, without qualification that it is mercenary to marry a nurse is simply silly; what I did say was that the high percentage of marriages of our nurses might very well be due to the fact that having shown they were women of a thrifty nature by the very fact of their joining the Fund they would have an extra attraction in the eyes of mercenary men.

### Appointments.

Belfast Union Infirmary. — Miss Anna Wilhelmina Graham has been appointed matron. She was trained at Belfast Union Infirmary and Fever Hospital. She has since been charge nurse at the Belfast Union Infirmary, superintendent nurse at Larne, assistant superintendent at Belfast Union Infirmary, and superintendent of the lunatic and convalescent department at Belfast Workhouse. She holds the certificate of the Ulster Obstetric Society.

Chorlton Union.—Miss Elizabeth Maude Smith has been appointed hospital matron. She was trained at St. Bartholomew's Hospital, London, and has since been junior and senior assistant matron of the Poplar and Stepney Sick Asylum, Bromley, E.

Coventry and Warwickshire Hospital.—Miss C. Hoadley has been appointed matron. She was trained at Guy's Hospital, London, and has since been matron of Goole Cottage Hospital, assistant matron at the Royal Infirmary, Preston, and lady superintendent at the Royal National Hospital for Consumption for Ireland, Newcastle, County Wicklow.

Fever Hospital, Johnstone, Renfrewshire. — Miss Florentia Daley has been appointed charge nurse. She was trained at Sunderland Fever Hospital, and has since been charge nurse at Tamworth Isolation Hospital. She has also done private nursing.

Hackney Union Infirmary.—Miss Edith Ventour has been appointed charge nurse. She was trained at Lambeth Union Infirmary, and has been nurse at Cheltenham Hospital.

Maidstone Union.—Miss May Minnie Whissel has been appointed charge nurse. She was trained at St. Marylebone Infirmary, and has since been staff nurse at the Cottage Hospital, Margate.

Northampton General Hospital.—Miss Annie Densham has been appointed matron. She was trained at the Leeds General Infirmary, where she has since been night superintendent and assistant matron.

Nottingham Children's Hospital.—Miss K. Maud Moore has been appointed lady superintendent. She was trained at St. Bartholomew's Hospital, London, and has since been ward sister at the Hospital for Sick Children, Great Ormond Street, London; matron of the convalescent branch of that institution, and matron of the Royal Hospital for Children and Women, Waterloo Bridge Road, London.

Oxford Union Infirmary.—Miss Annie MacSweeney has been appointed assistant nurse. She was trained by the Meath Workhouse Nursing Association, at the Children's Hospital, Temple Street, Dublin.

Perth District Asylum, Murthly.—Miss J. McLeod has been appointed matron. She was trained at Stirling District Asylum, Larbert, and Aberdeen Royal Infirmary, and has since been assistant matron at Gartloch Asylum.

Portsmouth Parish Infirmary.—Miss Gertrude Scott has been appointed night charge nurse. She was trained at Durham County Hospital, and has had experience in Plymouth Homœopathic Hospital, and has also been sister at the Portsmouth Parish Infirmary.

### Death in Our Ranks.

On April 29th Miss Florence Lew, since 1896 one of the staff of Guy's Trained Nurses' Institution, died at Guy's Hospital, after an illness of six weeks' duration, most patiently and cheerfully borne.

### TRAVEL NOTES AND QUERIES.

By Our Travel Correspondent.

In the Highlands (Nurse X. Y.)—I do not think you could do better than Boat of Garten, under the Cairngorm Mountains. It is on the Highland line between Perth and Inverness, not far from Killiecrankie and Pitlochrie, etc. . . . The place itself is not very beautiful, but the air is remarkably fresh and invigorating, and it cannot be yet much tourist ridden, as there is, I believe still but one hotel. I stayed there three years ago, but cannot remember the name. It does not matter, however; address the Proprietor, Hotel by the Station, Boat of Garten. I was very comfortable, and the terms were about what you wish, rather lower than higher. It is a very good centre for excursions. There are endless charming drives and walks, and the village itself is on a moor covered with heather. You see the Pass of Braemar in front. As a rule, the inland scenery is finest in Scotland on account of the mountains. Boat of Garten being on the Highland line, you are able to make excursions easily and cheaply in all directions. Another lovely spot is North Ballachulish, near to the Pass of Glencoe. There the country is ideally lovely, but it is not so bracing as Boat of Garten. However, if you like to try a few days there, address Mr. Carmichael, Temperance Hotel, North Ballachulish. It is on the Caledonian Canal. To reach it from Boat of Garten, you would need to go to Inverness for one night, and take the steamer from there. So glad my recommendation as to Cornwall was satisfactory. I felt sure you would like that hotel.

Preliminary Notice of another Fortnight in Switzerland.—It has been suggested to me that I should take another party to Switzerland this summer, and after the success of last year's tour, I have decided to again visit the Bernese Oberland, if a party is formed. We shall divide our time between Wengen and Grindelwald. Wengen is 4,000 feet above the sea, commands a splendid view of the snow-capped Jungfrau, and overlooks the picturesque Lauterbrunnen valley. Grindelwald, over 3,000 feet high, is the centre of some of the most magnificent scenery in Switzerland. Mürren, the famous Staubbach waterfall, the Great Scheidegg, the Little Scheidegg, the Faulhorn, and the Upper and Lower Grindelwald Glaciers, are some of the many spots whi h can be easily visited in a day's excursion from either Wengen or Grindelwald. The holiday will be organised for a fortnight, and the cost will be about *ten guineas.* (This includes board and lodging at very comfortable hotels, and second-class travelling from London.) The tickets will be available for 25 days for those who care to prolong their visit. The outward journey will probably be *viâ* Dover, Calais, Laon, and Interlaken, and the homeward one *viâ* Interlaken, Lake Brienz, the Brünig Pass, Lucerne, and Paris, so that those who wish can stay at Lucerne and Paris on the way back. We shall probably start on Tuesday, August 2nd. I shall be assisted by one or more colleagues, and one of us will return at the end of the fortnight with those who desire to be conducted on the homeward journey. It will be advisable to apply early as the hotels wish to know before the end of June how many of us are to be accommodated, Switzerland being exceedingly full and the hotels crowded during the summer months. Further details will be sent on application to L. Edna Walter, B.Sc., A.C.G.I., Inspector to the Board of Education, Secondary Branch, 38 Woodberry Grove, Finsbury Park, London, N.

### Echoes from the Outside World.

### The King and Queen in Ireland.

The great event of the visit of the King and Queen to Dublin was the ceremony of laying the first stone of the Royal College of Science at Leinster Lacon, in the heart of the city. There was an imposing Royal procession through the principal streets, and a considerable military display. The Royal procession was loudly cheered along the route. In the evening of the same day their Majesties witnessed at the Theatre Royal an excellent performance by Mr. Tree's company, at the close of which "God Save the King," started faintly at first, was sung with enthusiasm. The King and Queen left Dublin on Saturday for Kilkenny Castle. An address was presented by the Kilkenny Corporation, and in the course of his reply the King said that the Queen and himself have the fullest sympathy with the efforts for the revival of Irish industries, and will rejoice to find Ireland taking that place in the industrial world which the intelligence, the deftness, and the artistic tastes of her people amply qualify her to fill. In the afternoon their Majesties visited the Agricultural Show, and there was a great popular demonstration on the ground, as well as along the route. The city was beautifully illuminated in the evening. On Sunday morning the King and Queen attended service at the Cathedral. On Monday they visited Waterford quite unexpectedly, and had an extremely enthusiastic reception. In the course of one of his replies to the various addresses presented to him his Majesty said that he should welcome an industrial revival in Waterford as throughout the country which would give full scope at home to those native energies which have done so much to enrich other lands. As at Kilkenny, the King and Queen visited the spring show of the local agricultural society, and, also as at Kilkenny, the city was brilliantly illuminated at night. On Tuesday the King and Queen spent a quiet time at the Duke of Devonshire's seat at Lismore. The Queen took some photographs, and the Princess Victoria indulged in her favourite pastime of fishing. In the afternoon there was a motor-car tour from Lismore to the plain of Tipperary. On Wednesday morning their Majesties planted a cedar on the lawn at Lismore Castle, the visit to Ireland terminating in the afternoon.

### The War in the Far East.

According to the official accounts from Tokyo of the sinking of a Japanese transport, when the ship, struck by a torpedo, broke in two, the soldiers rushed up on deck and poured volleys from their rifles into the *Rossiya*. As the vessel was disappearing beneath the waves several soldiers were seen to commit suicide. A circular has been issued by the Russian Government denying that proposals of intervention have been made to Russia by any foreign Power. Russia, it is declared, did not want war, and everything within the limits of possibility was done to solve in a peaceful manner the complications which had arisen in the Far East. But Russia being forced to take up arms, no friendly mediation can have any success. Neither, it is added, will the Russian Government admit the intervention of any Power in the negotiations with Japan after the termination of hostile operations. Heavy fighting has taken place on the Ya-lu. The Japanese began the attack on the Russian positions on Tuesday last week. On Thursday they established themselves on the right bank of the river, and having in the course of Saturday constructed two pontoon bridges, two divisions crossed during the night, and a general attack was begun at dawn on Sunday. The Japanese forces captured Kiu-lien-cheng, which is regarded as the key of Russia's position on the right bank of the river. Official reports from Tokyo state that the Russians were defeated with heavy loss, while 28 of their guns were captured. Their casualties are said to amount to more than 800, two generals being wounded, and those of the Japanese to about the same number.

### The New Master of the Temple.

The appointment of the new Master of the Temple brings from a country rectory in Hertfordshire to London a former President of Trinity College, Oxford. The Rev. Henry George Woods, who succeeds the late Dr. Ainger, has been rector of Little Gaddesdon since 1898. He is 62 years of age, and commenced his clerical life, after a distinguished career at Oxford, in 1866. From 1872 till 1890 he was morning preacher at St. Nicholas, Abingden, but most of his time has been spent at Oxford. Dr. Woods has also for some years been chaplain and librarian at Ashbridge to Lord Brownlow. Mrs. Woods is one of the daughters of the late Dean Bradley, of Westminster, and has written a dramatic poem called "A Princess of Hanover."

### Pictures at the Royal Academy.

One of the strongest pictures in the Academy is by a woman. Miss Lucy Kemp-Welch in her "Timber Hauling in the New Forest" has drawn a group of brawny horses, who are palpably straining their muscles and throwing all their strength into their task of dragging the felled wood up a grassy slope, the surrounding oaks, as yet untouched by the woodman's axe, forming a suggestive contrast. Places of honour also belong to Mr. Herbert Draper's "The Golden Fleece," which is a striking example of well-harmonised colour, and shows the Greek heroes slaving at the oar, and to Mr. David Farquharson's "Full Moon and Spring Tide," representing a wild north coast and a long stretch of water on which the moon is throwing its weird lights and shadows. "The Golden Dawn" of Mr. Walter Donne, a view from the steep hill town of Roquebrune, near Mentone, is another much-talked-of, much-praised canvas. There are several noticeable religious pictures Mr. Solomon contributes "An Allegory," a picture of a dead Christ borne aloft by angels. Mr. Sigismund Goetz' "Despised and Rejected of Men" portrays the dead Christ tied up to an altar around which life goes on unheeding. All classes pass by, the newsboy, flower-seller, gilded youth, society lady, sportsman, priest, and scientist. The only person who turns round and looks towards the Saviour as if she understood the call is a hospital nurse. Mr. Robert Sauber has a gigantic picture called "Mammon," and Mr. Abbey has a Crucifixion painting on panel which is worth careful study. Amongst the portraits "Mrs. Werthheimer," by Mr. Sargent, is a masterpiece.

### Funeral of Miss Farren.

The late Miss Nellie Farren (Mrs. Soutar) was buried on Monday at Brompton Cemetery, followed by many friends, theatrical and otherwise, and attended by a dense crowd of people. The grave is near the centre of the cemetery, and, notwithstanding the pouring rain, people, holding dripping umbrellas and standing in pools of water, lined the approaches three or four deep. Miss Farren was the daughter of Henry Farren, actor and manager. Her first appearance was as the little Duke of York in "Richard III." at the age of seven; her next in the pantomime at the theatre in the New Cut at the age of 15. Four years later she joined the Gaiety company, where she and her husband acted for 25 years. One of her greatest successes was in "Little Dr. Faust," which the artist Whistler went to see over fifty times. In 1891 Miss Farren left the stage owing to ill health, and was an invalid till her death. In 1898 a performance was arranged for her benefit at which £6,000 was raised and an annuity purchased. Now that she no longer requires the money, £1,000 will be devoted to the establishment of a "Nellie Farren" cot in a children's hospital.

### A Book and its Story.

NEW BOOK BY THE AUTHOR OF "ELIZABETH AND HER GERMAN GARDEN."

Every admirer of books by this gifted writer will welcome a new one from her pen. "Elizabeth," we may remark in passing, is not to be confused with the author of "The Visits of Elizabeth," and other books concerning her. She is an English lady, who having married into a German family of distinction has, since then, been domiciled in the country, which so far forms the background of all her writings. In "Elizabeth in Rügen" we find the charming characteristics which have made the author's previous books so attractive. There is the same dainty humour and delightful play of fancy, the same refinement of style, and exquisite impressionism; and behind all, there lies the magnetism of a personality, infinite in variety, which gives an added grace to every scene and character described in a book written in the first person.

The reader is at once introduced to Rügen geographically, for in the first paragraph of the opening chapter we read: "Everyone who has been to school, and still remembers what he was taught there, knows that Rügen is the biggest island Germany possesses, and that it lies in the Baltic off the coast of Pomerania." Elizabeth has a wise preference for walking, in place of any other mode of moving, as being the most leisurely and delightful one for those observers of nature who wish to see into the life of things. "It is the one way of freedom. If you go to a place on anything but your own feet, you are taken there too fast, and miss a thousand delicate joys waiting for you by the wayside. . . . Successively did I invite the most likely of my women friends, numbering at least a dozen, to walk with me. They one and all replied that it would make them tired, and that it would be dull. . . . Walking, then, was out of the question, for I could not walk alone. The grim monster Conventionality, whose iron claws are for ever on my back, for ever pulling me back from the harmless and the wholesome, put a stop to that, even if I had not been afraid of tramps, which I was." The decision to make Rügen the point of attraction for a brief change of scene had been arrived at by Elizabeth having accidentally come upon Marianne North's, "Recollections of a Happy Life," when turning over the books one hot afternoon in the library at home. The description she gave of Rügen and its attractions, backed by that found in an old guide to Rügen, containing the following quaint paragraph, made her hasten her preparations for departure:

"Hearest thou the name of Rugen, so doth a wondrous spell come over thee. Before thine eyes it rises as a dream of far-away beauteous fairylands. Images and figures of long ago beckon thee across to the marvellous places where in grey prehistoric times they dwelt, and on which they have left the shadow of their presence. And in thee stirs a mighty desire to wander over the glorious, legend-surrounded island. Cord up, then, thy light bundle, take to heart Shylock's advice to put money in thy purse, and follow me without fear of the threatening sea-sickness which may overtake thee on the short crossing, for it has never yet done anyone any more harm than imposing on him a rapidly passing discomfort." It did not take Elizabeth long, she tells us, to make up her mind, still less to cord up her light bundle, for somebody else did that, and two days after she had read Marianne North's description of a sandy cove where the water was always calm, where bathers floated about on its crystal surface, and the beautiful jelly-fish, stars of purest colours, floated with them, she and Gertrud, her German maid, got out of a suffocating train into the freshness that blows round ryefields near the sea, and began their journey into the unknown. Elizabeth being unable, for lack of a companion, to carry out her desire to walk through Rügen, had taken her own carriage and manservant. "The carriage was a light one of the Victoria genus, with a hood; the horses were a pair esteemed at home for their meekness; the coachman, August, was a youth who had never yet driven straight on for an indefinite period without turning round once, and he looked as if he thought he were going to enjoy himself. The carriage had been sent on the day before, and round it on our arrival stood the station authorities in an interested group. Evidently the coachman had told them what I was going to do, and I suppose such an enterprising woman does not get out at Miltzow every day." Packed in with the greatest care, there only remained to stow away the various odds and ends necessary to every woman, who in travelling has an eye to comfort as well as utility. "In the hood were the umbrellas, guide-books, and maps, besides one of those round, shiny, yellow wooden band-boxes into which every decent German woman puts her best hat. This luggage and some mysterious bundles on the box that the coachman thought were hidden by his legs, but bulged out unhideable on either side, prevented our looking elegant; but I did not want to look elegant, and I had gathered from remarks of those who had refused to walk that Rügen was not a place where I should meet anyone who did." Freed from the fetters of conventionality, Elizabeth's spirits rose in proportion. The fields were whitening to the harvest; overhead the sky was a hot blue. The road lay through an undulating country; in the distance, on either side, lay farmhouses sheltering in the hollows, their red roofs visible at intervals, and "high above our heads the larks quivered in the light, shaking out that rapturous song that I can never hear without a throb of gratitude for being alive."

Among the quaint places visited in Rügen each with a lively description of its attractions, Elizabeth went to Vilm, a spot much frequented by artists. "If you love out-of-door beauty, wide stretches of sea and sky, mighty beeches, dense bracken, meadows radiant with flowers, chalky levels purple with gentians, solitude, and economy, go and spend a summer at Vilm. The inn is kept by one of Prince Putbus's foresters, or rather by his amiable and obliging wife, the forester's functions being apparently restricted to standing picturesquely propped against a tree in the front of the house in a nice green shooting suit, with a telescope at his eye through which he studies the approaching, or departing launch. His wife does not rest. The Frau Förster insisted on showing me the bedrooms. They are very simple and very clean, each one with a beautiful view. The rest of the house, including the dining-room, does not lend itself to enthusiastic description. I saw the long table at which the four and twenty painters eat. They were doing it when I looked in, and had been doing it the whole time I was under the chestnuts. There were at least forty people learning to be patient, and one waiter and a boy to drive the lesson home."

Elizabeth in her lonely rambles through the woods, or by the shore, had one congenial companion in a much-loved book "Wordsworth's Prelude." "I brought it because I know of no other book that is at the same time so slender and so satisfying. It slips even into a woman's pocket and has an extraordinary effect on the mind. You must to enjoy it be first a lover of Wordsworth. You must love the uninspired poems for the sake of the divineness of the inspired poems." There were unexpected *rencontres* in this out of the world corner, and these, with persons who played a part in them, are sketched with an inimitable grace and humour. "Elizabeth in Rügen" is an altogether charming book.

"Elizabeth in Rügen." By the author of "Elizabeth and Her German Garden," etc. (Macmillan & Co. 6s.)

### Notes and Queries.

**FOR REGULATIONS SEE PAGE 12.**

*L.O.S.*

(37) Please explain the meaning of L.O.S., and also of "massage and electricity combined."—*Anxious.*

L.O.S. stands for the London Obstetrical Society; these letters being used by persons to denote that they hold the Society's certificate for midwifery. "Massage and electricity combined" signifies that the masseuse understands how to carry out treatment by electricity in connection with massage.

*Certificate of Maternity Nursing.*

(38) Can you tell me how to obtain a certificate for maternity nursing? I was trained at a large general hospital abroad, and for six months at Plaistow, E., and have nine years' experience, but I have not certificates to show for it, only testimonials from medical men.—*Perplexed.*

You should apply to the Secretary, the Central Midwives Board, 6 Suffolk Street, Pall Mall, and ask if your qualifications are sufficient for your name to be put on the Roll of Registered Midwives.

I have a certificate for four months' maternity training and have had four months' training at the Women's Hospital, Sparkhill, and seven years' experience. I should be glad to know if my qualifications will fit me for the new registration certificate.—*Nurse E. M.*

See reply to *Perplexed.*

Will you kindly tell me where I should apply to be registered as "maternity nurse" under the Midwives Act?—*E. A.*

If by maternity nurse you mean "midwife," apply to the Secretary, the Central Midwives Board, 6 Suffolk Street, Pall Mall, S.W.

*Nurses over 40.*

(39) Can you tell me of a well-known co-operative institute for nurses over 40?—*A. L. R.*

The only society of the kind is for the older members of the Royal British Nurses' Association, for particulars apply to the Secretary, the Auxiliary Nurses' Society, 10 Orchard Street, Portman Square, W.

*Recognised Midwifery Schools.*

(40) Which are the recognised training schools for midwifery not including Queen Charlotte's Hospital?—*Urgent.*

Apply for advice to the Secretary, the Association for Promoting the Training and Supply of Midwives, 12 Buckingham Street, Strand, W.C.

*Nursing at Ely.*

(41) Can you let me know what nursing institutions or hospitals there are in Ely, and if it would be wise to start private nursing single-handed there?—*Nurse S.*

The only institution for the relief of the sick in Ely is the City of Ely Dispensary, St. Mary's Street. We can offer no opinion as to a nurse's prospects, which could only be ascertained by local inquiries.

*Government Appointments.*

(42) Are there any hospital appointments under Government, excepting in Army hospitals, open to other than Army nurses? What is the age limit? Are there any general hospitals, or hospitals for Women under Government in South Africa? With the view of obtaining any of these appointments, to whom should a nurse apply?—*Mona.*

Apply to the Colonial Nursing Association, Imperial Institute, S.W., where you will obtain full details as to nursing in the Colonies, either under Government or independently.

**Important Nursing Textbooks.**

"Nursing Profession: How and Where to Train." 2s. net; 2s. 4d. post free.

"Nurses' Dictionary of Medical Terms and Nursing Treatment." By Honnor Morten. 2s. post free.

"Nursing." By Isabel Hampton. 7s. 6d. net; 7s. 10d. post free.

"Surgical Ward-Work and Nursing." By Dr. Alex. Miles. 3s. 6d. net; 3s. 10d. post free.

"Outlines of Insanity." By F. H. Walmsley, M.D. Popular Edition. 1s. 6d. post free.

"Notes on Pharmacy and Dispensing for Nurses." By C. J. S. Thomson. 1s. post free.

The above works are obtainable of all booksellers, or direct from The Scientific Press, Limited, 28 & 29 Southampton Street, Strand, London, W.C.

### For Reading to the Sick.

ASCENSIONTIDE.

Thou art gone up on high,
But Thou didst first come down
Through earth's most bitter misery
To pass unto Thy crown;
And girt with griefs and fears
Our onward course must be;
But only let this path of tears
Bring us at last to Thee.

Thou art gone up on high,
But Thou shalt come again
With all the bright ones of the sky
Attendant in Thy train.
Lord, by Thy saving power
So make us live and die
That we may stand in that dread hour
At Thy right hand on high.

*E. Toke.*

Ascension Day especially puts before us our Lord in His human nature, because it was in that nature that He ascended up to Heaven. "Thou madest Him lower than the angels, to crown Him with glory and worship: Thou madest Him to have dominion over the works of Thine hands, and hast put all things in subjection under His feet." So was it accomplished on the day when our Lord, even as the Apostles beheld Him, "was taken up and received into heaven, and sat on the right hand of God." "Lift up your heads, O ye gates; and be ye lift up, ye everlasting doors; and the King of Glory shall come in." Let us worship Him in that seat in heaven; let us worship Him as Judge and Intercessor. As Judge, who sees into all hearts; and as Intercessor, Who pleads our cause. Let us worship Him with fear and love, remembering both His insight into us, and His compassion for us. He pleads, as perfect and sinless man, as the Second Adam, for the whole of the fallen race of Adam. He is our merciful and faithful High Priest; knowing our infirmities, inasmuch as He Himself has had experience of them; and knowing the strength of our temptations, inasmuch as He Himself also was tempted. Our Lord was perfect at each stage of His humanity; though, until His Ascension, His human nature did not enter into its fulness of glory in union with God. He gradually advanced towards this ultimate perfection; and the endurance of suffering under temptation was the appointed means through which His human nature passed on to this fulness. He has endowed us with grace and the gift of righteousness and has opened the door of Heaven. These are his gifts. Let us show our thankfulness for them by raising our hearts in prayer. By endeavouring to live as citizens of Heaven and as risen with Christ seeking those things which are above where Christ sitteth on the right hand of God.—*Mozley.*

O Saviour who for men has trod
The winepress of the wrath of God
Ascend and claim again on high
Thy glory left for us to die.

O Christ our Lord of Thy dear care
Thy lowly members heavenward bear
Be ours with Thee to suffer pain
With Thee for evermore to reign.

*From the Latin.*

